# Importance of respiratory syncytial virus as a predictor of hospital length of stay in bronchiolitis

**DOI:** 10.12688/f1000research.40670.2

**Published:** 2021-07-02

**Authors:** Jefferson Antonio Buendia, Diana Guerrero Patino

**Affiliations:** 1 Pharmacology and Toxicology Department, Pharmacology and Toxicology Research Group, Faculty of Medicine, Universidad de Antioquia., Medellín, Antioquia, 053212, Colombia; 2 Hospital Infantil Concejo de Medellín, Medellin, Antioquia, 055420, Colombia

**Keywords:** Bronchiolitis, Colombia, respiratory syncytial virus, length of hospital stay, chest indrawing

## Abstract

**Introduction** **:** Bronchiolitis is the leading cause of hospitalization in children. Estimate potentially preventable variables that impact the length of hospital stay are a priority to reduce the costs associated with this disease. This study aims to identify clinical variables associated with length of hospital stay of bronchiolitis in children in a tropical middle-income country

**Methods:** We conducted a retrospective cohort study in 417 infants with bronchiolitis in tertiary centers in Colombia. All medical records of all patients admitted to the emergency department were reviewed. To identify factors independently associated we use negative binomial regression model, to estimate incidence rate ratios (IRR) and adjust for potential confounding variables

**Results** **:** The median of the length of hospital stay was 3.68 days, with a range of 0.74 days to 29 days, 138 (33.17%) of patients have a hospital stay of 5 or more days. After modeling and controlling for potential confounders age <6 months, comorbidities (CHD or neurological), BPD,  chest indrawing, RSV isolation, and C-reactive protein were independent predictors of LOS

**Conclusions** **:** Our results show that in infants with bronchiolitis, RSV isolation, age <6 months, comorbidities (CHD or neurological), BPD,  chest indrawing, and C-reactive protein were independent predictors of LOS. As a potentially modifiable risk factor, efforts to reduce the probability of RSV infection can reduce the high medical cost associates with prolonged LOS in bronchiolitis.

## Introduction

Bronchiolitis is the most frequent lower respiratory tract infection in infants ^1, 2^. One of the variables with more incidence in the financial burden of this disease is the hospital length of stay (LOS). Among inpatients with bronchiolitis, approximately a quarter undergo a prolonged length of stay (LOS) ^3^. The hight medical cost associates with prolonged LOS in bronchiolitis imposes an economic burden, especially in tropical middle-income countries ^4^. LOS is a direct measure of the quality of health service ^5^.

Some models have identified predictors of LOS such as age, underlying conditions (congenital heart disease, chronic lung conditions, immunocompromised states), low weight, male gender, clinical characteristics at admission, prematurity, RSV isolation ^6^. However, many of these models lack accuracy ^7^ or were made in patients without significant comorbidities ^8^. Otherwise, in tropical areas in addition to genetic differences, the respiratory syncytial virus (RSV), generates differences in the burden of morbidity and mortality given the non-seasonality of these areas ^9^. In this context, there is a critical need to explore predictors of LOS, especially in tropical areas, improving their accuracy of current models .This information will allow risk management for healthcare and prioritize care strategies in groups with a high probability of prolonged hospital stay to reduce their impact on hospital costs and morbidity. This study aims to identify clinical variables associated with LOS of bronchiolitis in children in a tropical middle-income country.

## Methods

We conducted a retrospective cohort study that included all infants with bronchiolitis younger than two years of age in tertiary centers in Rionegro, Colombia, from January 2019 to December 2019. The municipality of Rionegro had a total population of 101,046 inhabitants, with two tertiary referral hospitals ^10^. Inclusion criteria were defined as children younger than two years of age admitted to the pediatric ward diagnosed with bronchiolitis, according to the national clinical guideline of bronchiolitis (first wheezing episode younger than 24 months of age) ^11^. Patients without lower respiratory compromise, with positive bacterial cultures on admission, confirmed whooping cough (culture or PCR) were excluded. The study protocol was reviewed and approved by the Institutional Review Board of the University of Antioquia (No 18/2015).Informed consent was obtained from all parents or caregivers of the patients included in the study, following the clinical research standards in Colombia, and prior approval by the ethics committee.

### Procedures

 We collected the following variables: age, sex, weight, height, signs, and symptoms on admission (including fever, chest indrawing, chest auscultation, %SpO _2_), vaccination scheduled chart for age, exposure to cigarette smoking, history of prematurity and bronchopulmonary dysplasia confirmed by a neonatologist at the time of discharge from the NICU, comorbidities (congenital heart disease, neurological disease), diagnostic tools as chest X rays, hemograms, etc. Additionally, we collected variables related to outcomes of care or disease-severity parameters such as length of hospital stay. In our hospitals, bronchodilators and systemic steroids are used at the discretion of attending physicians according to national clinical guidelines of bronchiolitis ^11^. Nasopharyngeal aspirate (NPA) was taken immediately upon admission to the emergency department within 48 hrs of admission using standardize technique. RSV was confirmed using direct immunofluorescence (Light Diagnostics TM Respiratory Panel 1 DFA, Merck-Millipore Laboratory).NPA data for other viruses were no available in our institution consistently. 

### Statistical analysis

Continuous variables were presented as mean ± standard deviation (SD) or median (interquartile range [IQR]), whichever appropriate. Categorical variables are shown as numbers (percentage). Differences between continuous variables were analyzed using the unpaired *t*-test or Wilcoxon's signed-rank test, whichever was appropriate. Associations between categorical variables and the outcome variable were analyzed using the chi-square test or Fisher's exact test, as needed. To identify factors independently associated with length of hospital stay, we used a Poisson regression model, or negative binomial regression model in case of the presence of overdispersed count data, to estimate incidence rate ratios (IRR) and adjust for potential confounding variables. We only include initially variables associated with LOS with values of p <0.2 or that change the effect estimate by more than 10% after their inclusion. The variable selection and modeling processes were made following the recommendations of Greenland ^12^. The goodness of fit of the model was evaluated using Hosmer–Lemeshow test and area under curve in Poisson regression or Akaike information criterion (AIC), Bayesian information criterion (BIC) in negative binomial regression. All statistical tests were two-tailed, and the significance level used was *p* < 0.05. The data were analyzed with Stata v15.0 (Stata Corporation, College Station, TX).

## Results

### Study population

During the study period, 417 cases of bronchiolitis were included. A total of 66% of the patient was less than 6 month, most of them males (60%), with supportive O _2_ (83%). RSV was isolated in 200 patients (48%). Of these, 81 patients had a history of premature birth and 17 of them with BPD. A total of 20 patients had some cardiac or neurological disease and 10 of them with a history of use of palivizumab. Table 1 presents the clinical characteristics of the population. Deidentified individual-level raw data are available from Zenodo ^13^.

**Table 1.** Demographic features and clinical information of the patients included in the study.

Variable	n (%)
Age less than 6 month	277(66.43)
Male, n(%)	251(60.34)
Premature birth	81(19.47)
Comorbidities (CHD or neurological)	20(4.81)
BPD	17(4.09)
Atopy	17(4.09)
Previously hospitalization by bronchiolitis	30(7.21)
Exposure to cigarette smoking	49 (11.9)
Exclusive maternal breastfeeding for at least six month	102(24.4)
%SpO2, median(ds)	89(0.28)
O2 supportive, n(%)	347(83.41)
Clinical & laboratory parameters	
Fever	119(28.61)
Chest indrawing	184(44.23)
Tachypnea	48(13.30)
Rhonchi	137(32.93)
Crepitation	137(32.93)
Abnormal X-ray *	109(26.33)
Leucocytosis (> 15.000/mm3)	51(12.26)
RSV positive	200(48.48)
Increased C-reactive protein (> 4 mg/lit.)	327(78.61)

*Atelectasis (n=7), alveolar(n=16) or interstitial (n=48) infiltrates, hyperinflation(n=38) CHD : Congenital heart disease, BPD: Bronchopulmonary dysplasia, RSV: Respiratory syncytial virus

The median of the length of hospital stay was 3.68 days, with a range of 0.74 days to 29 days and an interquartile range of 4.06 days. Among all 417 patients, 138 (33.17%) have a hospital stay of 5 or more days

### Multivariate analysis of predictors associated with LOS

Unavariate analysis is presented in Table 2. Due to the significative presence of overdispersed count data was detected (Likelihood-ratio test of alpha=0, χ ^2^= 203.97, p=0.000), a negative binomial regression model was used to adjust for potential confounding variables. The predictive variables included in the complete model were age, sex, premature birth, comorbidities, BPD, atopy, previously hospitalization by bronchiolitis, %SpO _2_, fever, signs of respiratory distress, RSV, Leucocytosis (>15.000/mm ^3^) and increased C-reactive protein (>4 mg/lit.). After modeling and controlling for potential confounders in the negative binomial regression: age <6 months, comorbidities (CHD or neurological), BPD, chest indrawing, RSV isolation, and C-reactive protein were independent predictors of LOS ( Table 3).

**Table 2.** Demographic features and clinical information of the patients included in the study.

Variable. n(%)	n (%)	Incidence rate- ratio (95% CI)	p
Age less than 6 month	277(66.43)	0.998 (0.997-0.998)	0.000
Male , n(%)	251(60.34)	0.048 (0.967-1.157)	0.218
Premature birth	81(19.47)	1.319 (1.191-1.461)	0.000
Comorbidities (CHD or neurological)	20(4.81)	1.787(1.525-2.094)	0.000
BPD	17(4.09)	1.037(0.835-1,289)	0.738
Atopy	17(4.09)	0.827(0.650-1.052)	0.123
Previously hospitalization by bronchiolitis	30(7.21)	0.750(0.619-0.908)	0.003
Exposure to cigarette smoking	49 (11.9)	1.040(0.910-1.188)	0.563
Exclusive maternal breastfeeding for at least six month	102(24.4)	0.627(0.550-1.012)	0.423
SpO2, median(ds)	89(0.28)	1.007(0.999-1.015)	0.055
O2 supportive , n(%)	347(83.41)	2.227(1.899-2.611)	0.000
Clinical & laboratory parameters			
Fever	119(28.61)	0.834(0.754-0.9232)	0.000
Chest indrawing	184(44.23)	1.416(1.297-1.545)	0.000
Tachypnea	48(13.30)	1.181(1.028-1.357)	0.018
Rhonchi	137(32.93)	0.777(0.704-0.856)	0.000
Crepitation	137(32.93)	1.088(0.984-1.182)	0.160
Abnormal X-ray *	109(26.33)	1.055(0.957-1.164)	0.277
Leucocytosis (> 15.000/mm3)	51(12.26)	1.179 (1.040-1.337)	0.010
RSV positive	200(48.48)	1.653(1.511-1.807)	0.000
Increased C-reactive protein (> 4 mg/lit.)	327(78.61)	0.849(0.767-0.940)	0.002

*Atelectasis (n=7), alveolar(n=16) or interstitial (n=48) infiltrates, hyperinflation(n=38) CHD : Congenital heart disease, BPD: Bronchopulmonary dysplasia, RSV: Respiratory syncytial virus

**Table 3.** Multivariate analysis of predictors associated with length of stay.

	IRR	CI 95%	p
Age <6 months	0.998	0.998-0.999	0.000
Comorbidities (CHD or neurological)	2.119	1.459-3.078	0.000
Chest indrawing	1.322	1.115-1.567	0.001
BPD	1.610	1.087-2.385	0.017
RSV positive	1.593	1.346-1.886	0.000
C-reactive protein	1.005	1.002-1.008	0.006

## Discussion

The main purpose of this study was to determine the independent clinical variables associated with LOS of bronchiolitis in children in tropical middle-income countries. Our study shows that RSV, age <6 months, comorbidities (CHD or neurological), BPD, chest indrawing, and C-reactive protein were independent predictors of LOS

Our results emphasize the importance of knowing the presence of RSV. While some predictors of LOS, such as age, comorbidities, and potentially initial signs of respiratory distress, can not be modified, others as RSV isolation are potentially modifiable by interventions such as futures vaccines or palivizumab in a high-risk population. Previous studies in populations with seasonality had revealed the importance of RSV as a predictor of hospital stay. DeVicenzo *et al*., in a sample of 141 infants <24 months old without previous chronic cardiac or lung disease or prematurity, in Tennessee found that higher nasal RSV load was an independent predictor of longer hospitalization. A 1-log higher RSV load predicted a 0.8-day longer hospitalization, reflects the higher RSV load that occur earlier in the disease ^14^. Mansbasch *et al*., in a prospective cohort of 2207 infants of 16 US hospital without excluding patients previous chronic cardiac or lung disease or prematurity, also found that patients with RSV have a higher proportion of patient with prolonged LOS (>3 days) than patients with only HSV infection, but less than RSV+HRV co-infection (48% vs 28% vs 54%, p<0.001 ^15^. Rodríguez-Martínez, in 303 infants with acute bronchiolitis in Bogota, also found that RSV isolation correlated with a hospital stay of 5 or more days (OR 1.92, CI 95% 1.02 to 3.73) ^3^. In Qatar, Janahi *et al*., , detected RSV in 51.2% of in 369 patients admitted to the pediatric ward for bronchiolitis, but no association was found between RSV and LOS ^16^. Additionally, Masarweh *et al*., in a retrospective study of 4793 infants with bronchiolitis in a single tertiary medical center in Israel between 2001–2009, found that RSV isolation did not correlate with LOS ^17^. In this evidence, only the Mansbach study used the PCR assay for viral detection, but the results with immunofluorescence assay with respect to the predictive value of RSV were similar to the PCR assay. Indeed, the main problem of the studies mentioned above was the serious statistical mistakes of analyzing the LOS. While we used a negative binomial regression model, due to the presence of overdispersed count data, to adjust for potential confounding variables to analyze LOS, studies by Rodriguez, Devicenzo, and Mansbach dichotomize the LOS to perform logistic regression, while Masarweh's study performed a linear regression; being both approaches not completely correct. The loss of information from dichotomizing a continuous outcome is well documented in the literature, and even worse, analyzing a variable that does not have a normal distribution with a linear regression invalidates this method of analysis ^18^. These pitfalls in statistical analysis can explain the lack of accuracy of predictive models ^6^. The regression models recommended are median, gamma, or Poisson regression; which have some type I error but avoid the mistakes previously mentioned with the logistic or linear regression model.

Othe variable potentially modifiable associated with LOS was age <6 months. Our findings are consistent with previous results reported in the literature and provide further evidence that younger infants are at a greater risk of requiring prolonged LOS ^3^. This can be explained because the smaller caliber of the airways in younger infants and poor innate immune response to RSV in newborns, making younger infants more susceptible to severe forms of viral infections and prolonged LOS ^19, 20^. Preventive strategies such as the use of palivizumab in a high-risk population or the use of future vaccines that confer immunity in children under 6 months against RSV; will constitute possibly effective interventions in reducing the economic burden of this disease.

Several predictive models had reports consistently the chest indrawing as predictive of prolonged LOS that is which is biologically plausible and expected due that this sign also is a universal marker of severity of the disease, as well as the presence of underlying conditions (congenital heart disease, chronic lung conditions, immunocompromised states) ^3, 6– 8, 21– 23^ or C-reactive protein (CRP) as a biomarker of severity and bacterial co-infection in patients hospitalized for bronchiolitis ^24– 26^

Our study has limitations. First, since this study was based on medical records review, we cannot include other variables such as environmental pollution and genetic factors, and residual confounding cannot be excluded. Second, respiratory syncytial virus was confirmed using direct immunofluorescence, which may underestimate the real burden of viral infection. However, despite this possible underestimation, RSV infection was positively associated, and it is possible that the magnitude of the IRR is even greater. The detection of other respiratory viruses was not homogeneous in all patients, so to avoid information bias we decided not to include them in the analysis. We cannot rule out that other respiratory viruses have equal or greater association with our dependent variable in the study. Third, the study was conducted in a tertiary referral hospital, and therefore the patients included represent the high spectrum of severity, limiting the generalization of results to other contexts. However, the similarity of our population in terms of clinical characteristics, risk factors, and seasonality of bronchiolitis in our country with previous reports suggest strength and consistency in our results ^3, 4^

## Conclusion

Our results show that in infants with bronchiolitis, RSV, age <6 months, comorbidities (CHD or neurological), BPD, chest indrawing, and C-reactive protein were independent predictors of LOS in a tropical middle-income country. As a potentially modifiable risk factor, efforts to reduce the probability of RSV infection can reduce the hight medical cost associates with prolonged LOS in bronchiolitis.

## Data availability

### Underlying data

Zenodo: Importance of respiratory syncytial virus as a predictor of hospital length of stay in Bronchiolitis. http://doi.org/10.5281/zenodo.4432434 ^13^.

This project contains the raw data for each patient assessed in the present study.

Data are available under the terms of the Creative Commons Attribution 4.0 International license (CC-BY 4.0).

## Declarations

### Ethics approval

The study protocol was reviewed and approved by the Institutional Review Board of Clinica Somer (No 281015) and the University of Antioquia (No 18/2015).

### Consent for publication

All authors consent this paper for publication

## Abbreviations

incidence rate ratios (IRR)

hospital length of stay (LOS)

respiratory syncytial virus (RSV)

Nasopharyngeal aspirate (NPA

Bronchopulmonary dysplasia (BPD)

Chronic heart disease (CHD)
