# Supplementary material for: Importance of respiratory syncytial virus as a predictor of hospital length of stay in bronchiolitis
Source: F1000Res. 2022 Jul 8;10:110. Originally published 2021 Feb 15. [Version 4] doi: 10.12688/f1000research.40670.4 (PMC9277196; doi:10.12688/f1000research.40670.4)
Supplement: Peer review history

# Peer review: Reviewer response for version 4

**DOI:** 10.5256/f1000research.135025.r143683

**Published:** 2022-07-12

**Authors:** Joan L Robinson

**Affiliations:** 1 Department of Pediatrics, University of Alberta, Edmonton, Alberta, Canada

One residual problem is that it is not clear what (ds) means in Table 2.

Is the work clearly and accurately presented and does it cite the current literature?

Partly

If applicable, is the statistical analysis and its interpretation appropriate?

I cannot comment. A qualified statistician is required.

Are all the source data underlying the results available to ensure full reproducibility?

Yes

Is the study design appropriate and is the work technically sound?

Yes

Are the conclusions drawn adequately supported by the results?

Yes

Are sufficient details of methods and analysis provided to allow replication by others?

Yes

Reviewer Expertise:

Pediatric infectious diseases

I confirm that I have read this submission and believe that I have an appropriate level of expertise to confirm that it is of an acceptable scientific standard.

# Peer review: Reviewer response for version 3

**DOI:** 10.5256/f1000research.81423.r116157

**Published:** 2021-12-24

**Authors:** Joan L Robinson

**Affiliations:** 1 Department of Pediatrics, University of Alberta, Edmonton, Alberta, Canada

I am not very happy with the response to my review. The authors simply put the word "corrected" after each of my comments even though some of the comments were not addressed. For example, the discussion still talks about HSV. The authors should tell the reviewer how they addressed each comment. 

 A CRP of 4 mg/L is completely normal. The authors can still claim that a higher CRP predicted a longer LOS but they need to note that this was not necessarily an elevated CRP.

Is the work clearly and accurately presented and does it cite the current literature?

Partly

If applicable, is the statistical analysis and its interpretation appropriate?

I cannot comment. A qualified statistician is required.

Are all the source data underlying the results available to ensure full reproducibility?

Yes

Is the study design appropriate and is the work technically sound?

Yes

Are the conclusions drawn adequately supported by the results?

Yes

Are sufficient details of methods and analysis provided to allow replication by others?

Yes

Reviewer Expertise:

Pediatric infectious diseases

I confirm that I have read this submission and believe that I have an appropriate level of expertise to confirm that it is of an acceptable scientific standard, however I have significant reservations, as outlined above.

# Response

**Published:** 2021-12-25

**Authors:** Jefferson Buendia

Dear reviewer.

 You can see that all grammar suggestions were corrected according to your advice. 

 When we place in our corrections the word "corrected" it means that the text was changed as required by your advise. We do not consider the need to place the multiple grammar corrections in other text , when these can be see in the text with the track changes in word.

 The cut-off of the CPR estimated in our mathematical model associated with our outcome was 4 mg/L, and this just is the value of our paper. This variable was managed continuously and no preset cut-off point ( eg dichotomic , etc). As was stated in the discussion these values should be alert to the doctor in conjunction with other variables that may be is a patient with a longer hospital stay or seen another way patient with a lower value together with other variables can help to the pediatrician to predict the lack for examen of intensive use of resources by the patient not having a high risk of prolonged hospital staly

# Peer review: Reviewer response for version 2

**DOI:** 10.5256/f1000research.58307.r99869

**Published:** 2021-11-29

**Authors:** Joan L Robinson

**Affiliations:** 1 Department of Pediatrics, University of Alberta, Edmonton, Alberta, Canada

The authors analyzed data from all children less than 2 years of age admitted in 2019 to two hospitals in Columbia to establish factors identifiable at admission that predict a longer length of stay (LOS). The factors that they identified include RSV, age <6 months, comorbidities (CHD or neurological), BPD, chest indrawing, and a higher C-reactive protein.

 The main question is whether their findings have practical implications. Some of the risks that they identified are not well established (in particular the higher CRP). They make the point that identification of these risk factors for prolonged LOS may inform their palivizumab program and eventually an RSV immunization program. The risk factors that would be helpful for such programs are age < 6 months and comorbidities, both of which are well recognized as risk factors for RSV hospitalization but less so for LOS. Nonetheless, both are currently widely used in palivizumab programs worldwide.

 Minor points:

1. Abstract – What does “admitted to the emergency department” mean. Do the authors perhaps mean “admitted through the emergency department”?
2. “Among inpatients with bronchiolitis, approximately a quarter undergo a prolonged length of stay”. There is no standard definition of prolonged LOS, so it is difficult to know what the authors mean by this.
3. The high medical cost associates with prolonged LOS in bronchiolitis imposes an economic burden, especially in tropical middle-income countries.” Why? Surely it is even a greater burden in low income countries. I do not understand why it is a special problem in “tropical countries”.
4. “Some models have identified predictors of LOS such as age, underlying conditions (congenital heart disease, chronic lung conditions, immunocompromised states), low weight, male gender, clinical characteristics at admission, prematurity, RSV isolation” – I think that the authors mean “low birthweight”. Throughout the paper, I do not know what they mean by “RSV isolation” as almost no centers do viral culture anymore. They need to change it to “detection of RSV”.
5. “Otherwise, in tropical areas in addition to genetic differences, the respiratory syncytial virus (RSV), generates differences in the burden of morbidity and mortality given the non-seasonality of these areas”  - I do not know what this sentence means.
6. By “in tertiary centers”, do the authors mean “admitted to tertiary centers”? Patients can also be in the emergency department of a hospital.
7. Do the national guidelines encourage or discourage use of bronchodilators and systemic steroids? I think that most discourage both these days.
8. Please reword “NPA data for other viruses were no available in our institution consistently.”  I think that the authors mean “not” rather than “no”.  
9. In Table 1, are these parameters at admission? For example, “%SpO2, median(ds)”, is this the value on admission without oxygen? What does “ds” mean?
10. Throughout the paper, I would round all percentages to the nearest whole number or provide only one decimal point. It makes it easier for the reader and the sample size does not justify two decimal points.
11. Table 3 and the abstract need to specify which CRP was considered to be significant.
12. The DeVicenzo study described a longer LOS with a higher amount of RSV in secretions. This is quite different from showing that RSV itself increases the LOS.
13. “Mansbasch *et al*., in a prospective cohort of 2207 infants of 16 US hospital without excluding patients previous chronic cardiac or lung disease or prematurity, also found that patients with RSV have a higher proportion of patient with prolonged LOS (>3 days) than patients with only HSV infection, but less than RSV+HRV co-infection (48% vs 28% vs 54%, p<0.001” – I do not understand why HSV would be compared to RSV.

Is the work clearly and accurately presented and does it cite the current literature?

Partly

If applicable, is the statistical analysis and its interpretation appropriate?

I cannot comment. A qualified statistician is required.

Are all the source data underlying the results available to ensure full reproducibility?

Yes

Is the study design appropriate and is the work technically sound?

Yes

Are the conclusions drawn adequately supported by the results?

Yes

Are sufficient details of methods and analysis provided to allow replication by others?

Yes

Reviewer Expertise:

pediatric infectious diseases

I confirm that I have read this submission and believe that I have an appropriate level of expertise to confirm that it is of an acceptable scientific standard, however I have significant reservations, as outlined above.

# Response

**Published:** 2021-12-12

**Authors:** Jefferson Buendia

**Response : Reviewer 2**

- *The authors analyzed data from all children less than 2 years of age admitted in 2019 to two hospitals in Columbia to establish factors identifiable at admission that predict a longer length of stay (LOS). The factors that they identified include RSV, age <6 months, comorbidities (CHD or neurological), BPD, chest indrawing, and a higher C-reactive protein. The main question is whether their findings have practical implications. Some of the risks that they identified are not well established (in particular the higher CRP). They make the point that identification of these risk factors for prolonged LOS may inform their palivizumab program and eventually an RSV immunization program. The risk factors that would be helpful for such programs are age < 6 months and comorbidities, both of which are well recognized as risk factors for RSV hospitalization but less so for LOS. Nonetheless, both are currently widely used in palivizumab programs worldwide.*
- **Response:** Corrected. It was clarified in the document and in the tables the term (CRP level of more than  4 mg/lit) .
- *Abstract – What does “admitted to the emergency department” mean. Do the authors perhaps mean “admitted through the emergency department”*
- **Response:** Corrected.
- *“Among inpatients with bronchiolitis, approximately a quarter undergo a prolonged length of stay”. There is no standard definition of prolonged LOS, so it is difficult to know what the authors mean by this.*
- **Response: **Corrected.
- *The high medical cost associates with prolonged LOS in bronchiolitis imposes an economic burden, especially in tropical middle-income countries.” Why? Surely it is even a greater burden in low income countries. I do not understand why it is a special problem in “tropical countries”.*
- **Response: **Corrected.
- *“Some models have identified predictors of LOS such as age, underlying conditions (congenital heart disease, chronic lung conditions, immunocompromised states), low weight, male gender, clinical characteristics at admission, prematurity, RSV isolation” – I think that the authors mean “low birthweight”. Throughout the paper, I do not know what they mean by “RSV isolation” as almost no centers do viral culture anymore. They need to change it to “detection of RSV”.*
- **Response: **Corrected.
- *“Otherwise, in tropical areas in addition to genetic differences, the respiratory syncytial virus (RSV), generates differences in the burden of morbidity and mortality given the non-seasonality of these areas”  - I do not know what this sentence means.*
- **Response: **Corrected.
- *By “in tertiary centers”, do the authors mean “admitted to tertiary centers”? Patients can also be in the emergency department of a hospital.*
- **Response: **Corrected.
- *Do the national guidelines encourage or discourage use of bronchodilators and systemic steroids? I think that most discourage both these days.*
- **Response: **deleted this sentence.
- *Please reword “NPA data for other viruses were no available in our institution consistently.”  I think that the authors mean “not” rather than “no”.  *
- **Response:** Corrected.
- *In Table 1, are these parameters at admission? For example, “%SpO2, median(ds)”, is this the value on admission without oxygen? What does “ds” mean?*
- **Response: **Corrected.
- *Throughout the paper, I would round all percentages to the nearest whole number or provide only one decimal point. It makes it easier for the reader and the sample size does not justify two decimal points.*
- **Response: **Corrected.
- *Table 3 and the abstract need to specify which CRP was considered to be significant.*
- **Response: **Corrected.
- *The DeVicenzo study described a longer LOS with a higher amount of RSV in secretions. This is quite different from showing that RSV itself increases the LOS.*
- **Response:** Corrected.
- *“Mansbasch et al., in a prospective cohort of 2207 infants of 16 US hospital without excluding patients previous chronic cardiac or lung disease or prematurity, also found that patients with RSV have a higher proportion of patient with prolonged LOS (>3 days) than patients with only HSV infection, but less than RSV+HRV co-infection (48% vs 28% vs 54%, p<0.001” – I do not understand why HSV would be compared to RSV.*
- **Response:** Corrected.

# Peer review: Reviewer response for version 2

**DOI:** 10.5256/f1000research.58307.r88922

**Published:** 2021-11-12

**Authors:** Mauricio T. Caballero

**Affiliations:** 1 INFANT Foundation, Buenos Aires, Argentina

The authors have resolved the suggestions made.

Is the work clearly and accurately presented and does it cite the current literature?

Yes

If applicable, is the statistical analysis and its interpretation appropriate?

Partly

Are all the source data underlying the results available to ensure full reproducibility?

Yes

Is the study design appropriate and is the work technically sound?

Yes

Are the conclusions drawn adequately supported by the results?

Partly

Are sufficient details of methods and analysis provided to allow replication by others?

No

Reviewer Expertise:

Respiratory virus infection in children.

I confirm that I have read this submission and believe that I have an appropriate level of expertise to confirm that it is of an acceptable scientific standard.

# Peer review: Reviewer response for version 1

**DOI:** 10.5256/f1000research.43735.r85075

**Published:** 2021-06-28

**Authors:** Mauricio T. Caballero

**Affiliations:** 1 INFANT Foundation, Buenos Aires, Argentina

Importance of respiratory syncytial virus as a predictor of hospital length of stay in bronchiolitis is an interesting retrospective study conducted in tertiary centers in Rionegro, Colombia, from January 2019 to December 2019. The study explores variables associated with length of stay due to bronchiolitis in children under two years old. I have few comments regarding the study methods and results.

1. Respiratory syncytial virus was confirmed using direct immunofluorescence, which may underestimate the real burden of viral infection. How do authors estimate this could impact the results?
2. Authors did not mention if other respiratory viruses were explored and compared as associated to length of hospital stay. 
3. Univariable analysis should be shown in an extra table.

Is the work clearly and accurately presented and does it cite the current literature?

Yes

If applicable, is the statistical analysis and its interpretation appropriate?

Partly

Are all the source data underlying the results available to ensure full reproducibility?

Yes

Is the study design appropriate and is the work technically sound?

Yes

Are the conclusions drawn adequately supported by the results?

Partly

Are sufficient details of methods and analysis provided to allow replication by others?

No

Reviewer Expertise:

Respiratory virus infection in children.

I confirm that I have read this submission and believe that I have an appropriate level of expertise to confirm that it is of an acceptable scientific standard, however I have significant reservations, as outlined above.

# Response

**Published:** 2021-06-30

**Authors:** Jefferson Buendia

We appreciate your valuable comments. 

- *Respiratory syncytial virus was confirmed using direct immunofluorescence, which may underestimate the real burden of viral infection. How do authors estimate this could impact the results?*
- **Response:** Corrected. This limitation was added to the discussion of the paper. 

- *Authors did not mention if other respiratory viruses were explored and compared as associated to length of hospital stay.*
- **Response: **Corrected. This limitation was added to the discussion of the paper. 

- *Univariable analysis should be shown in an extra table.*
- **Response:** Corrected. Added a new table with the univariate analysis. 
